# A Look to the Future: Potential Theranostic Applications in Head and Neck Tumors

**DOI:** 10.3390/cancers17040695

**Published:** 2025-02-19

**Authors:** Jorge D. Oldan, Lilja B. Solnes, Bennett B. Chin, Steven P. Rowe

**Affiliations:** 1 Molecular Imaging and Therapeutics, Department of Radiology, University of North Carolina, Chapel Hill, NC 27599, USA; 2 The Russell H. Morgan Department of Radiology, Johns Hopkins University School of Medicine, Baltimore, MD 21287, USA; lsolnes1@jhmi.edu; 3 Department of Radiology, University of Colorado Anschutz Medical Campus, Boulder, CO 80045, USA; bennett.chin@cuanschutz.edu

**Keywords:** theranostics, head and neck, PET, DOTATATE, PSMA, FAP, CAIX

## Abstract

Theranostics, the practice of using a diagnostic radiopharmaceutical to guide radiotherapy with a chemically identical (or nearly identical) therapeutic radiopharmaceutical, is an exciting new field under development within the auspices of nuclear medicine. We aim to provide a narrative review of the areas of theranostics use and development which are occurring specifically in the head and neck, with attention to the therapeutic use of existing diagnostically used radiotracers such as agents that target prostate-specific membrane antigen (PSMA) and somatostatin receptors (e.g., DOTATATE derivatives), as well as developing classes of radiotracers such as those targeting fibrinogen-activating protein (FAP) and carbonic anhydrase IX (CAIX), as well as new *meta*-iodo-benzylguanidine (MIBG) derivatives. Most of those agents are still at the preclinical or early clinical stages of development, but as this is an emerging field, we aim to both outline current progress and suggest future directions.

## Simple Summary

Theranostics, which means using one radioactive drug to decide whether a related radioactive drug with a similar chemical shape is useful, is a growing field in finding and treating cancer. We review a number of these pairs of drugs specifically being studied for tumors of the head and neck and describe the clinical studies on patients that have been conducted already for each drug pair. We also discuss drugs that have not been tested in human patients yet to give an idea of the future of the field.

## 1. Introduction

The idea of theranostics is that the same (or a very similar) scaffold that is of diagnostic use may also be of therapeutic use by replacing a gamma- or positron-emitting diagnostic radionuclide with a chemically similar beta- or alpha-emitting therapeutic radionuclide. While something of a “buzzword” in recent times, the principle actually dates back to 1936 with the use of iodine-131 sodium iodide as a therapeutic [1], which was followed by various radionuclides for skeletal metastases beginning in the 1950s. Eventually, other targeted radiotracers for diagnostic imaging were leveraged with their theranostic “twins”, such as iodine-131 *m*-iodobenzylguanidine (^131^I-MIBG) for neural crest tumors in the 1980s. The actual term, “theranostics”, appeared in the 1990s with the proposal of the somatostatin model, with the diagnostic positron emission tomography (PET) agent ^68^Ga-DOTATATE being U.S. Food and Drug Administration (FDA)-approved in 2016, followed by the companion therapeutic, ^177^Lu-DOTATATE (Lutathera) in 2018 [1].

One challenge in the head and neck specifically is that by far the most common head and neck malignancy, squamous cell carcinoma, does not have a specific agent targeted to it, meaning that there remains significant work to be conducted on the preclinical side to delineate targets and appropriate imaging and therapeutic molecules. The use of radioiodine for well-differentiated thyroid cancers has been well described elsewhere and is discussed in another manuscript in this special issue.

However, other specialized diagnostic radiotracers, such as those targeting somatostatin receptors (such as DOTATATE) or the prostate-specific membrane antigen (PSMA) such as ^68^Ga-PSMA-11 or ^18^F-DCFPyL/piflufolastat F 18, have been converted into therapeutic agents, and these have begun to be explored in the head and neck. MIBG analogs is well explored, and ^131I^-MIBG has been used in multiple contexts. A variety of new radiotracers are under development as well, such as fibrinogen-activating protein (FAP) inhibitors and binders, carbonic anhydrase IX (CAIX) inhibitors and monoclonal antibodies, and CXCR4-targeting agents [2]. Explorations of theranostics using modifications of some of these agents have been performed as well, though often not in the head and neck.

In this review, we (1) describe the promising targets in head and neck cancer that might be leveraged for theranostic applications, (2) discuss the potential limitations of current imaging approaches, and (3) provide insight into the applicability of future agents. An overview of agents is provided in Table 1; an overview of the existing data (including case reports) for the efficacy of each theranostic is provided in Table 2.

## 2. 2-Deoxy-2-[^18^F]fluoro-D-glucose (FDG)

The most common diagnostic agent used in PET, 2-deoxy-2-[^18^F]fluoro-D-glucose (FDG), is a poor choice for theranostics for a number of reasons. While there are therapeutic radiohalogens that can be used to create radiotracers that utilize similar radiochemistry [35], those radionuclides tend to be much larger than fluorine-18 and would perturb the nature of some small molecules such as FDG. In addition, no specific agent targeted at glucose transporters currently exists [36]. One way around this would be using Cerenkov radiation from FDG to activate doxorubicin for chemotherapy [37]; in vitro proof-of-concept studies have explored that [37], but further development has not been published as of yet.

## 3. Prostate-Specific Membrane Antigen (PSMA)

It has been known since the first scans that PSMA-based agents, while targeted at cancer of the prostate, are taken up heavily in the salivary glands, and this is a source of the sialotoxicity of some therapeutic agents (as high as three-quarters with some 225Ac agents) [38]. Recently, attempts have been made to utilize this to treat salivary tumors (usually adenoid cystic carcinoma or salivary duct cancer) with PSMA-targeted theranostic agents [39], particularly given the success of ^177^Lu-PSMA-617 for prostate cancer in the VISION [40] and TheraP trials [41]. There have been early trials suggesting a dramatic response [3], stabilization of disease and pain control [4,5], or at least pain reduction [6] in isolated cases (usually 1–6 patients). However, at least one trial found insufficient tumor doses to continue [7], and the largest trial (15 patients) showed stable disease in less than a third of patients with adenoid cystic carcinoma and none in salivary duct carcinoma [8]. A number of clinical trials have been started to investigate the theranostic potential of PSMA-targeted agents in adenoid cystic carcinoma, but none have reported findings as of yet [39].

Some thyroid cancers express PSMA as well [39,42]. However, there seems to be significant heterogeneity among lesions in terms of uptake of radioiodine, FDG uptake, and PSMA-targeted radiotracer uptake [43]. Radioactive therapy of well-differentiated thyroid cancer usually begins (and often ends) with radioiodine, but in iodine-refractory cases, other tracers are often explored. PET in refractory iodine-avid tumors shows detection rates from 25 to 100%, but generally less than FDG [42]. There were two trials of ^177^Lu-PSMA in three patients [9,10], with mixed results. In one study, five patients with widely metastatic thyroid cancer were scanned, finding lesions not seen on FDG-PET, two were treated, and one showed improvement (the other progressed) [9]. The other was a trial on a single patient after tyrosine kinase inhibitors failed; the patient showed temporary improvement but ultimately progressed [10]. In both cases, there was significant uptake in tumors before treatment.

Endolymphatic sac tumors have been shown to have uptake with PSMA-targeted radiotracers and ^68^Ga-DOTATATE [44], but therapeutic applications have yet to be explored. Nonetheless, those findings hint at the overall big picture. Just as with chemotherapeutic regimens with targeted agents, we are moving into an era where the expression of an appropriate target for targeted radioligand therapy may be more important than the cell of origin.

## 4. Somatostatin Receptor Inhibitors (DOTATATE)

**Figure 1:**
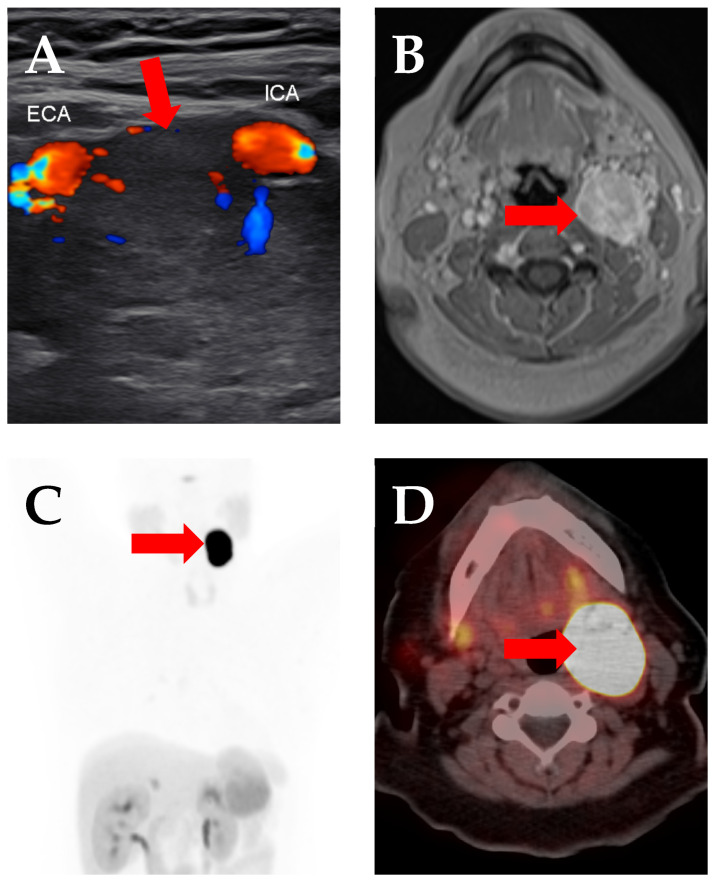
Images from a 46-year-old woman with a carotid body tumor. (**A**) Transverse ultrasound image demonstrates a heterogeneously hypoechoic mass (arrow) that is splaying the external carotid artery (ECA) and the internal carotid artery (ICA). (**B**) The mass was also imaged with axial, T1, contrast-enhanced MRI, where the significant vascularity of the tumor was reflected in avid enhancement (arrow). (**C**) Maximum intensity projection ^68^Ga-DOTATATE PET and (**D**) axial ^68^Ga-DOTATATE PET/CT images show intense uptake in the mass, suggesting a potential role for theranostics in such tumors.

Paragangliomas, including head and neck paragangliomas, take up radiotracers targeted against the somatostatin receptor, such as labeled peptides based on DOTATATE (Figure 1), which has already shown success in treating midgut neuroendocrine tumors in the NETTER-1 trial that led to FDA approval of ^177^Lu-DOTATATE [45]. Multiple cases have been treated with ^90^Y-DOTATATE or, more commonly, ^177^Lu-DOTATATE, usually with some mixture of stabilization of disease and partial response. Of those, the largest, and also most heterogeneous study, was a retrospective evaluation of 30 patients with pheochromocytoma and paraganglioma, 18 of whom had head and neck paragangliomas, treated with a mixture of ^177^Lu- and ^90^Y-DOTATATE or DOTATOC. That study showed 63% stable disease and 20% partial response [11]. A study of 14 patients treated with ^177^Lu-DOTATATE showed some decrease in uptake in 10 of 14 patients, with the best results in jugulotympanic paraganglioma [12]. Another study, which consisted of nine patients with inoperable head and neck paragangliomas treated with ^90^Y-, or a mixture of ^90^Y- and ^177^Lu-labeled DOTATATE, showed at least stable disease in all patients (four partial response (PR), five stable disease (SD)) [13]. A study of seven patients with head and neck paragangliomas (three carotid, three jugulotympanic, one combination) treated with ^177^Lu-DOTATATE showed at least stable disease in all and a decrease in tumor volume in four [14]. One study of four patients with neck and mediastinal paraganglioma treated with ^177^Lu-DOTATATE showed stable or partial response in all [15]. There is one case report of a complete response of a carotid body paraganglioma to ^177^Lu-DOTATATE [16] and another of stable disease with ^90^Y- and ^177^Lu-DOTATATE [17]. Initial results are thus promising, though a larger clinical trial is necessary.

**Figure 2:**
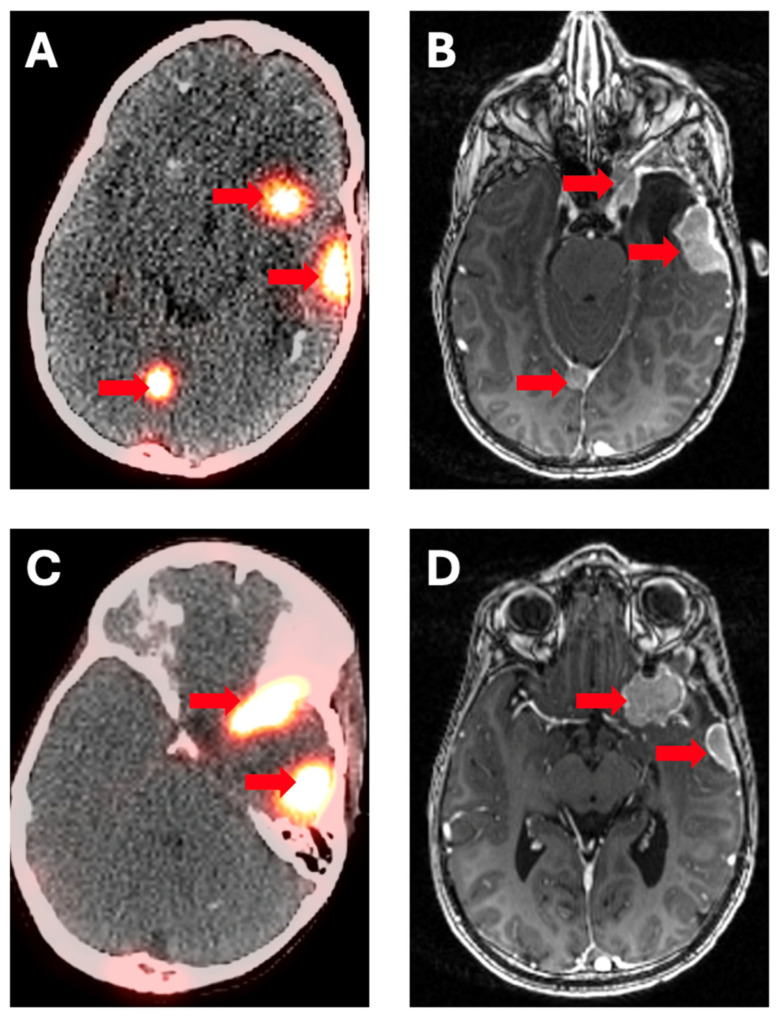
Images from a 48-year-old woman with multi-focal meningiomas. (**A**,**C**) Axial ^68^Ga-DOTATATE PET/CT images demonstrate tense uptake at multiple intracranial sites (arrows) that correspond to enhancing mass lesions on (**B**,**D**) contrast-enhanced, post-contrast, T1-weighted MRI (arrows).

Meningiomas have been known to express somatostatin receptors since the days of Octreoscan, and also unsurprisingly take up DOTATATE (Figure 2); interest in ^177^Lu-DOTATATE for progressive, treatment-resistant meningioma has existed for a few decades [46], with uptake correlating with receptor expression [47] and possibly negatively with metabolic rate [48], as with many other tumors. Early case reports show stable disease in the short term [18,19] and diagnosis of the occasional lung metastasis [49], mixed with the occasional lack of efficacy [20]. A number of small studies have at least shown the reasonable possibility of disease control. One study of 20 patients with unresectable, progressive meningioma treated with ^177^Lu-DOTATATE showed stable disease in half, predominantly in patients with higher ^68^Ga-DOTATATE uptake [21]; another of four showed stable disease in half [22]. A phase I clinical trial (NCT 03971461) of 14 patients with progressive meningiomas again showed stable disease in half for at least 6 months [23]; there is a phase II study (NCT04082520) ongoing. Another study with extended (median 5-year) follow-up of 42 patients mostly treated with ^177^Lu-DOTATATE (a few received ^90^Y-DOTATOC) had a 57% disease control rate, median PFS of 16 months, and median OS of 36 months; 6 were retreated with PFS and OS of 6.5 and 17 months, respectively [24].

There is some suggestion, starting with an early case report [25], of intraarterial administration succeeding where intravenous failed; a later study of eight patients treated both intravenously and intraarterially showed better dose and retention time by intraarterial administration, stable disease in seven of eight at 4 weeks and median time to progression of 8.9 months [26]; similarly, a later study of thirteen patients undergoing intraarterial therapy had eight with stable disease, one with partial remission, and one with complete remission [27]. It is thus possible intraarterial administration may have a better chance of achieving disease control.

## 5. Fibroblast-Activating Protein (FAP)

Fibroblast-activating protein (FAP) is the target of a new series of agents, most of them inhibitors (i.e., FAPI agents), such as ^68^Ga-FAPI-04 [50] and ^68^Ga-FAPI-46 [51]. Non-inhibitor molecules, such as peptide ^68^Ga-FAP-2286, are also under investigation and have shown promising results in certain cancer types [52]. While PSMA and DOTATATE bind specific receptors on the tumor itself (thus being theoretically more specific as they only bind to tumors with that receptor), FAP-targeted agents respond to the tumor microenvironment and bind to the cancer-associated fibroblasts (CAFs) surrounding many tumors. Those agents have already shown significant promise in the diagnostic space [53], with less background heart, liver, and brain uptake than FDG, the standard radiotracer for many tumor types with significant numbers of CAFs [54]. FAP-targeted agents may be more sensitive for bone metastases and radioiodine-negative thyroid cancers than FDG [54], as well as for gastrointestinal cancers [55]. However, as might be expected, they show uptake in fibrotic processes such as myelofibrosis, reactive nodes, arthritis, thyroiditis, and follicular thyroid adenoma, and the question of their effectiveness vis-à-vis FDG remains open [54]. There are also questions regarding the inter-reader reproducibility of these findings. One way or another, there has been increasing interest in them as radionuclide therapies.

While this remains very much at the research stage, there have been a few scattered cases of FAP-targeted agents being used in a theranostic capacity. For example, an agent with a related chemical structure (i.e., based on the (4-Quinolinoyl)-glycyl-2-cyanopyrrolidine scaffold [56]), ^177^Lu-DOTAGA.(SA.FAPi)2, has undergone a clinical trial of 15 patients with radioiodine-refractory and tyrosine kinase inhibitor-refractory differentiated thyroid cancer [28], with three patients having partial response and another four demonstrating stable disease (none showed complete response). It was also tried in one patient with medullary thyroid cancer, resulting in a decrease in the size of the neck mass with improvement of quality of life [29]. Another agent with the FAP-binding scaffold conjugated to ethylene blue (an albumin binder that increases circulation time), ^177^Lu-EB-FAPI (also known as ^177^Lu-LNC1004), was tested in a cohort of twelve patients with differentiated thyroid cancer and showed partial response in three patients and stable disease in another seven [30].

Among cases where the diagnostic radionuclide was switched out for a therapeutic analog, ^177^Lu-FAPI-46 has been described in multiple case reports. For example, in one case of differentiated thyroid cancer, a patient achieved stable disease [31]. Further, stable disease was also noted in a case of anaplastic thyroid cancer [32]; a mixed response was described in a case of metastatic nasopharyngeal cancer [33]; and symptomatic improvement was noted in a case of MEN 2A with medullary thyroid carcinoma as well as sacral paraganglioma and bilateral pheochromocytoma which did not take up radiolabeled DOTATATE or MIBG [34]. Dosimetry has been studied with the related agent, ^177^Lu-FAPI-04, in at least one patient with thyroid cancer [57], but efficacy has yet to be determined.

## 6. Carbonic Anhydrase IX (CAIX) Inhibitors

CAIX is a transmembrane enzyme that is often upregulated in hypoxic tumor microenvironments, as it regulates the acidity of tumor cells, but it can be constitutively expressed when there are mutations [58]; it also encourages metastatic properties such as decreased cell adhesion, activation of proteases, induction of vascularization, and enhancement of motility and migration [59]. High stromal expression of CAIX correlates negatively with survival [60]. It has been studied in head and neck extensively for assessment of hypoxia [61], including with humanized antibodies such as girentuximab [62] that are beginning to be explored therapeutically in cancers that express CAIX [63], such as renal cell carcinoma. Outside of direct theranostic applications, the role of hypoxia as a predictive and prognostic biomarker in patients with head and neck cancer suggests an important role for imaging of CAIX—i.e., that higher uptake of CAIX-targeted agents might be used to select appropriate therapeutic regimens and might also be negatively associated with patient outcomes. A mouse model in which CAIX-targeting has been leveraged to localize yttrium-90 and indium-111 to tumors has been explored [64]. However, theranostic applications in the head and neck have yet to be studied in humans.

## 7. CXCR4

While promising in hematopoietic malignancies such as lymphoma [65] and multiple myeloma and other diseases such as myelofibrosis and primary aldosteronism [66], agents targeted to CXCR4, most prominently ^68^Ga-pentixafor, are not well studied for head and neck cancers. The agent, however, has been studied for glioblastoma, with a database of ^68^Ga-pentixafor images of glioblastoma patients constructed [67]. At least one study of 19 patients shows that it is useful for assessing the presence of CXCR4 receptors in this tumor [68] and may have future potential in this area [69], with various peptides (most prominently 177Lu-Lu-DOTAT-POL3026) being shown to have activity against CXCR4-bearing tumor cells in vitro [70].

## 8. *meta*-I-benzylguanidine (MIBG) Derivatives

Replacing the diagnostic radionuclide iodine-123 with the therapeutic radionuclide iodine-131 on the adrenergic receptor-targeted compound MIBG has long been used in the treatment of metastatic pheochromocytoma and paraganglioma (which express adrenergic receptors). Indeed, high-specific-activity ^131^I-MIBG was the only United States Food and Drug Administration-approved agent for those indications [71] until it was recently withdrawn from the market.

In regards to head and neck paraganglioma, however, those tumors are actually commonly false-negative on MIBG scans [72], making adrenergic receptors a less attractive molecular target, with at least one purely diagnostic study of ^131^I-MIBG showing low sensitivity for head and neck paraganglioma [73]. Instead, such tumors often express somatostatin receptors and agents such as ^177^Lu-DOTATATE may be more worthwhile to explore and may also be significantly less toxic with fewer serious adverse events [74].

## 9. Discussion

The field of theranostics is quite active elsewhere in the body, with FDA-approved agents like 177Lu-DOTATATE and 177Lu-PSMA-617 being well-studied and in widespread use for neuroendocrine tumors [45] and prostate cancer [40,41]. Attempts to extend these agents to other tumors, specifically in the head and neck, have been much more limited in response, likely due to the lack of a good molecular target in most cases—the vast majority of head and neck cancers are aerodigestive tract squamous cell carcinomas, after all, for which no known specific target exists.

DOTATATE has shown the best results, likely due to the presence of SSTR receptors on paragangliomas just as on midgut neuroendocrine tumors. Early studies in tens of patients with head and neck paragangliomas do seem to show a mixture of partial response and stabilization of disease [11,12,13,45], similar to that on midgut tumors. Similarly, trials on progressive, treatment-resistant meningiomas show stabilization of disease in about half [21,23,24]; further trials are ongoing, and there is the possibility intraarterial administration of the drug may improve its efficacy.

The picture is unclear or less rosy for other groups of agents. For PSMA agents, there are case reports of initial success for adenoid cystic carcinoma, but early trials show stabilization of disease at best [7,8]. Use of these agents for iodine-refractory thyroid cancer similarly shows mixed results at best [9,10]. FAP agents show a mix of stabilization and partial response in differentiated thyroid cancer [28,29] and are under further study. CAIX and CXCR4 have not been studied in humans at the present time.

MIBG derivatives are a case where the theranostic agent is actually produced and FDA-approved but withdrawn from the market by the manufacturer due to limited usage and fixed costs. Head and neck paragangliomas, unlike other locations, are famously false-negative on MIBG scans [72], suggesting this may not be a promising molecular target in the head and neck, with DOTATATE above being preferred instead [74].

The toxicities of PSMA agents, in particular ^177^Lu-PSMA-617, are well described in the multiple trials that have been performed [40,41]. These include bone marrow suppression effects such as thrombocytopenia, anemia, and leukopenia, and decreased renal function, reaching Grade 3 or 4 in 5–15% of cases; salivary gland toxicity is also well documented. The toxicities of ^177^Lu-DOTATATE have similarly been explored in multiple large studies, with Grade 3–4 myelosuppression or decreased renal function being somewhat rarer (0–3%) but a carcinoid crisis occasionally occurring (<1%) and needing to be treated with SSTR antagonists. ^177^Lu-DOTATATE also requires simultaneous administration of amino acids over several hours to prevent nephrotoxicity [45]. Data for the other agents are much more limited, but a first-in-human, dose escalation FAP agent trial similarly found hematotoxicity to limit dose escalation in some patients [30].

## 10. Conclusions

In recent years, theranostic approaches in which targeted diagnostic and therapeutic radiotracers are combined to provide optimized precision medicine for patients have radically altered the treatment paradigms for patients with multiple different cancers [40,75]. The emergence of new radioligands, improvements in the availability and side-effect mitigation of α-particle emitting radionuclides, and the incorporation of new methods such as artificial intelligence into patient selection should all contribute to fulfilling the promise of the theranostic paradigm [76].

A number of promising new approaches outside of the imaging field are being developed. The theranostic genome, an exciting new listing of potential theranostic targets in the genome [77], offers the possibility of discovering a wealth of future new molecular targets, such as the mTOR pathway for photodynamic therapy [78].

Specifically, in regard to head and neck cancer, theranostics remains in its infancy. We have hints that DOTATATE-derived therapeutic agents may prove useful in head and neck paragangliomas and meningiomas, as they are in the remainder of the body, and that FAP-targeted agents may prove useful in multiple cancers, such as undifferentiated thyroid cancer. Applications of other molecular targets largely remain to be seen. Significant work remains—but the rich pipeline of new agents and targets should ensure a wealth of new information and opportunities for improvements in patient care.

## Figures and Tables

**Table 1:** Overview of agents. FDA-approved pharmaceuticals and uses are indicated by *; others are assumed experimental.

Target	Diagnostic Agent	Therapeutic Agent	Targets
PSMA	^18^F-flotufolastat *, ^18^F-piflufolastat *, ^68^Ga-PSMA-gozetotide *	^177^Lu-PSMA-617 * (vipivotide tetraxetan)	Prostate cancer *, adenoid cystic carcinoma, iodine-refractory well-differentiated thyroid cancer, endolymphatic sac tumor (under study)
DOTATATE	^68^Ga-DOTATATE (gozetotide) *, ^68^Ga-DOTATOC (edotreotide) *, ^64^Cu-DOTATATE *	^177^Lu-DOTATATE *, ^90^Y-DOTATATE	SSTR-positive neuroendocrine tumors (incl. gastroentericopancreatic, paraganglioma/pheochromocytoma) *, meningioma
FAP	^68^Ga-FAPI-04, ^68^Ga-FAPI-46, others	^177^Lu-DOTAGA.(SA.FAPi)2, ^177^Lu-EB-FAPI,	
CAIX	^89^Zr-girentuximab	None yet	Renal cell cancer
CXCR4	^68^Ga-pentixafor	^177^Lu-DOTAT-POL3026	Glioblastoma
Alpha2 adreno-ceptor	^123^I-MIBG *	^131^I-MIBG * (no longer made)	Neuroblastoma

**Table 2:** Efficacy and evidence for each tracer. ACC, adenoid cystic carcinoma, DTC, differentiated thyroid cancer; PD = progressive disease, SD = stable disease, PR = partial response, CR = complete response. FDA-approved pharmaceuticals and uses are indicated by *; others are assumed experimental.

Therapeutic Agent	Targets	n	Efficacy	Ref.
^177^Lu-PSMA-617 *	ACC	4	2 PR, 2 mixed	[3]
^177^Lu-PSMA-617 *	Salivary gland cancer (mixed)	6	1 SD, 1 PR; 4 pain red	[4]
^177^Lu-PSMA-617 *	ACC	1	SD, Pain reduction	[5]
^177^Lu-PSMA-617 *	ACC	1	PD, Pain reduction	[6]
^177^Lu-PSMA-617 *	Salivary gland cancer (mixed)	5	4 PD, 1 SD	[7]
^177^Lu-PSMA-617 *	ACC, salivary duct carcinoma	15	4 PD, 3 SD (5 discontinued tx)	[8]
^177^Lu-PSMA-617 *	Iodine-refractory DTC	2	1 PD, 1 PR	[9]
^177^Lu-PSMA-617 *	Iodine-refractory DTC	1	1 PD	[10]
^177^Lu-DOTATATE *, ^90^Y-DOTATATE, ^90^Y-DOTATOC	Paraganglioma/pheochromocytoma	30	4 PD, 19 SD, 7 PR	[11]
^177^Lu-DOTATATE *	HN Paraganglioma	14	10 PR (by SUV)	[12]
^177^Lu-DOTATATE *, ^90^Y-DOTATATE	HN paraganglioma	9	4 PR, 5 SD	[13]
^177^Lu-DOTATATE *	HN paraganglioma	7	4 PR, 3 SD	[14]
^177^Lu-DOTATATE *	HN/mediastinal paraganglioma	4	2 PR, 2 SD	[15]
^177^Lu-DOTATATE *	Carotid body paraganglioma	1	PR	[16]
^177^Lu-DOTATATE *, ^90^Y-DOTATATE	Carotid body paraganglioma	1	SD	[17]
^177^Lu-DOTATATE *	Meningioma (recurrent)	1	SD	[18]
^177^Lu-DOTATATE *	Meningioma (metastatic)	1	SD	[19]
^177^Lu-DOTATATE *	Meningioma (metastatic)	1	PD	[20]
^177^Lu-DOTATATE *	Meningioma (refractory, prog.)	20	10 PR, 10 SD	[21]
^177^Lu-DOTATATE *	Meningioma (progressive)	4	2 PD, 2 SD	[22]
^177^Lu-DOTATATE *	Meningioma (progressive)	14	7 PD, 7 SD	[23]
^177^Lu-DOTATATE *, ^90^Y-DOTATOC	Meningioma	42	23 SD, 1 PR	[24]
^177^Lu-DOTATATE * (IA)	Meningioma	1	1 PR	[25]
^177^Lu-DOTATATE * (IA)	Meningioma	8	1 PD, 7 SD	[26]
^177^Lu-DOTATATE * (IA)	Meningioma	13	3 PD, 8 SD, 1 PR, 1 CR	[27]
^177^Lu-DOTAGA.(SA.FAPi)2	RAI/TKI-refractory DTC	15	8 PD, 4 SD, 3 PR	[28]
^177^Lu-DOTAGA.(SA.FAPi)2	Medullary thyroid cancer	1	PR	[29]
^177^Lu-EB-FAPI	DTC	12	2 PD, 7 SD, 3 PR	[30]
^177^Lu-FAPI-46	DTC	1	SD	[31]
^177^Lu-FAPI-46	Nasopharyngeal cancer	1	SD	[32]
^177^Lu-FAPI-46	Nasopharyngeal cancer	1	Mixed response	[33]
^177^Lu-FAPI-46	MEN 2A (multiple cancers)	1	Symptomatic only	[34]
